# The effects of cognitive training on gait speed and stride variability in old adults: findings from a pilot study

**DOI:** 10.1007/s40520-014-0228-9

**Published:** 2014-05-07

**Authors:** Jean-Paul Steinmetz, Carine Federspiel

**Affiliations:** 1 Department of Research and Development, ZithaSenior, 30 rue Ste Zithe, L-2763 Luxembourg, Luxembourg; 2 Geriatric Rehabilitation Unit, ZithaKlinik, Luxembourg, Luxembourg

**Keywords:** Cognitive training, Gait speed, Gait stability, Dual tasking

## Abstract

**Background:**

The interrelationship between gait performance and higher-order cognitive functions has been established through a number of different investigations. In turn, enabling gait by improving cognition is a new and emerging field of research.

**Aims:**

Investigating if and to what extent a structured cognitive training program influences gait-related parameters in a sample of old and frail nursing home residents.

**Methods:**

Twenty-one nursing home residents were quasi-randomized to an intervention group following a 6-week structured cognitive training program or a control group. Gait was investigated during normal pace and under two dual-task conditions (simple and complex dual-task walking conditions), using the GAITRite^®^ system at three predefined time points (pre-intervention, post-intervention, 3-month follow-up). Outcome measures were gait speed and stride variabilities.

**Results:**

Confirmation of the interrelationship between gait and cognition evidenced by decreased gait parameters during complex dual-task walking. Observation of clinical meaningful improvements in gait stability and gait speed after the training program under the complex dual-task situations, with only speed remaining stable over a period of 3 months.

**Discussion:**

This study on the effects of cognitive training on gait is promising, with several results going in the expected direction. Our data corroborate previous findings and extend them to the group of frail old nursing home residents.

**Conclusions:**

The present pilot study’s approach of improving gait under challenging walking situations by interventions designed to improve cognitions adds encouraging results to this emerging field of research, although restrictions in sample size and in the control group prevent us from drawing firm conclusions.

## Introduction

In recent years, an increasing number of research studies focusing on the interplay between higher-order cognitive functions and gait in older adults suggest a causal link between cognitive dysfunctions, gait disorders and falls [1–5]. Age-associated degradation in motor functions is thought to be compensated by higher-order executive functions and attention. However and dramatically, when compensating for age-related motor declines older adults are facing a severe impasse as they have to rely on a deteriorating cognitive system [1].

Declines of gait and balance under dual-task (DT) situations have been observed in several studies, suggesting that walking is a complex motor task relying on high-level cognitive functions (for a review, see [6]). This view has been amplified by findings of a recent study, with participants experiencing alterations in almost all investigated gait measures (i.e., reduced velocity, increased variability) while walking and performing a second task, compared to a single walking condition [7]. In old adults, implications of higher cognitive function on gait seem, however, not to be restricted to a priori complex walking conditions. Data from Hausdorff and colleagues suggest that executive functions play a crucial role during the performance of normal and routine walking activities among older adults [8]. This leads to the conclusion that deficits in higher cognitive functions may result in an increased fall risk and hence, a compromised quality of life as was recently corroborated by research findings demonstrating that executive functions are associated with future falls for periods ranging up to 66 months [3, 4]. In sum, it is becoming largely accepted that gait is associated with higher-order cognitive functions in older adults [6, 8, 9]. Given this, a relatively new approach investigates if benefits of cognitive training programs can potentially transfer to untrained functional domains [10], such as gait. A study by Verghese [11] reports positive transfer effects from trained cognitive functions to untrained gait-related parameters. The authors found increased gait velocities compared to baseline measures in their intervention group (IG) following an 8-week training program (Cohen’s *d* = 0.44). No improvements were observed in the control group (CG) (*d* = 0.06). In another noteworthy study [12], positive effects of cognitive training on (instrumental) activities of daily living after a ten-year follow-up are reported. Hence and in sum, cognitive intervention programs on gait in older adults produced first results suggesting that cognitive interventions may enable old adults to successfully allocate their cognitive resources among two competing activities [3], thus improving their gait qualities. In the context of this emerging field, the present study therefore investigates if a previously validated cognitive intervention program [13] significantly improves gait parameters in frail old adults.

## Methods

### Participants and design

**Fig. 1:**
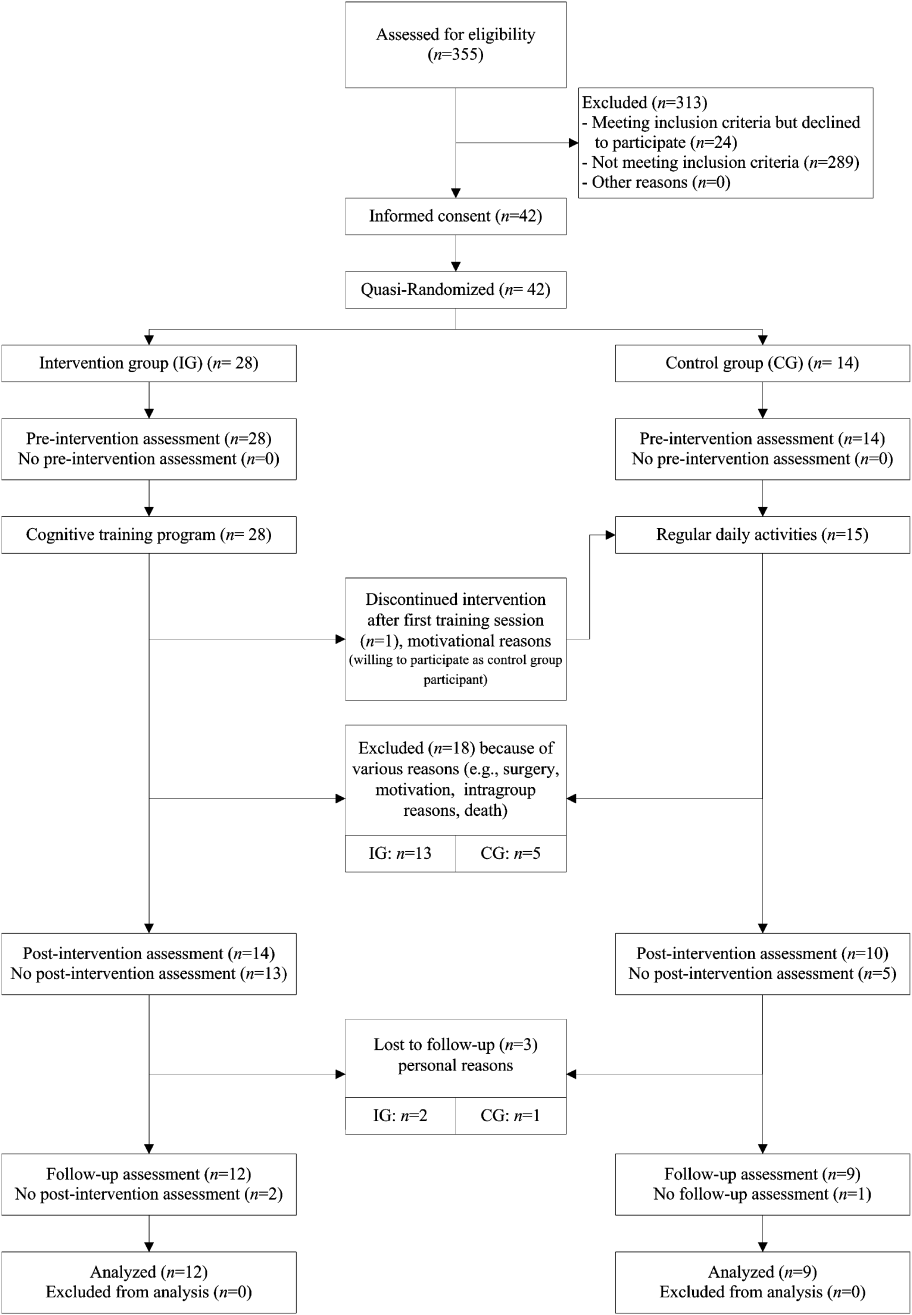
Diagram to show the flow of study participants

A total number of *N* = 355 nursing home residents were screened for eligibility. Eligibility criteria were a Mini Mental State Examination score ≥23, ability to walk without an ambulatory aid for more than 10 m and no substantial visual or hearing impairments unless corrected. Sixty-six individuals were identified meeting the inclusion criteria from which 42 provided informed consent. Our sample was quasi-randomized to two groups: one IG and one CG. Please refer to Fig. 1 for details on the flow of participants. Given the present applied research context and the pilot nature of the study, participants not willing to be attributed to the IG were asked to participate as CG participants in the study. We were unable to exclude participants demonstrating health concerns other than reduced cognitive functions. Participants were therefore screened for their frailty status (see Table 1 for a detailed overview). Gait parameter assessments were conducted in both study groups at three predefined moments: pre-intervention, post-intervention, and 3 months after the completion of the intervention by the IG (follow-up). Of the initial sample size of 42 participants in the pre-intervention assessment, drop-outs reduced the number of participants in both groups to *N* = 21 (12 IG and 9 CG; see Fig. 1). Only participants completing the three assessments (pre-intervention, post-intervention, follow-up) are included in the present analyses. The study was approved by the National Research Ethics Committee and written consent was obtained from all participants.

**Table 1:** Baseline characteristics

	Intervention (*n* = 12)	Control (*n* = 9)	*p* value (univariate)
Age, years	83.8 ± 6.5	85.7 ± 5.4	0.50
Body mass index	26.4 ± 5.5	25.5 ± 5.5	0.75
MMSE	27.1 ± 1.8	27.6 ± 2.0	0.58
Barthel	88.3 ± 13.7	95.6 ± 5.3	0.15
GDS-4	0.83 ± 0.72	0.44 ± 0.73	0.24
CIRS-G severity index	1.8 ± 0.45	2.0 ± 0.36	0.46
Get Up and Go Test, s	22.0 ± 9.4	19.3 ± 5.7	0.46
Grip strength maximal, kg	16.1 ± 4.1	19.8 ± 7.7	0.16
Falls in the last 6 months	0.82 ± 1.6	1.1 ± 1.6	0.69
Length of stay in facility, months	9.3 ± 9.4	51.3 ± 97.5	0.15
Participants, *n* (%)			
Females	10 (83.3)	7 (77.8)	0.59
Education			
<13 years	11 (91.7)	8 (88.9)	0.69
≥13 years	1 (8.3)	1 (11.1)	
Taking more than 6 drugs/day	11 (91.7)	8 (88.9)	0.69
With cardio-vascular drugs	7 (58.3)	5 (55.6)	0.62
With psychoactive drugs	10 (83.3)	8 (88.9)	0.61
Using a walking aid in everyday life	9 (75.0)	6 (66.7)	0.52
With fear of falling	6 (50.0)	3 (33.3)	0.38

All values are mean ± SD unless otherwise stated. GDS-4, Geriatric Depression Scale, 4 item version [17]; CIRS-G severity index, Cumulative Illness Rating Scale for Geriatrics [18]; severity index = (total CIRS-G score/total number of categories endorsed)

### Gait analysis

Gait analyses were performed according to the published European guidelines [14] using a 518-cm long GAITRite^®^ walkway (CIR Systems Inc., Clifton, NJ, USA). In total, three gait conditions were assessed, (a) normal walking, with participants instructed to walk at their habitual speed (single walking condition), (b) DT walking while counting out loud in an ascending order starting by 1 (simple DT condition), and (c) DT walking while counting backwards by twos out loud starting from a predefined number (complex DT condition). Under DT conditions, no explicit instructions on prioritization were given. Participants were instructed to perform two walks per condition, with the goal of increasing the number of steps to be analyzed and thus the precision of the measure. Gait parameters were collected without a walking aid [15], whereas participants were free to sit down and rest in between walks at their own convenience. The selection of gait parameters was limited to parameters that have previously been associated with gait dysfunctions and falls among old adults [7, 16]. Stride-to-stride variabilities are measured by the coefficient of variation [CoV = (standard deviation/mean) × 100]. For the present study, three gait parameters were retained for further analysis: velocity (cm/s), CoV of stride time (in %), CoV of stride length (in %).

### Cognitive training program

The cognitive training program [13] is composed of 12 training sessions to be administered in a period of 6 weeks (2 sessions per week). Sessions last 90 min each and are thoroughly structured and standardized. Training sessions were administered and supervised by experienced trainers. The participants of the IG were split up into smaller groups of three to five people; the rate of attendance of the IG participants over the 12 training sessions was 86.7 %. Each session was composed of five different types of content, (a) theoretical background on the topic of the day (i.e., meta-cognitive contents such as “how does my memory work”), (b) group exercises, (c) stimulating cognitive games, (d) individual exercises, and (e) home assignments. The cognitive abilities that were specifically trained via the training program are attentional capacities, working memory, the ability to plan, verbal fluency, learning and memory. The training program’s effectiveness in mild cognitive impairment was recently discussed [19].

### Statistical analyses

Statistical analyses are limited to a total of 21 participants (*n* = 12 in the IG and *n* = 9 in the CG). Prior to analyzing the data, outlier detection was conducted using the boxplot outlier labeling rule [20]. Distribution assumptions of the data were verified by examining distribution histograms and values of skewness, kurtosis and by using the Kolmogorov–Smirnov test. To investigate the influence of the two DT conditions on gait speed, CoV stride time and CoV stride length, repeated measures MANOVA was performed for the single walking, the simple DT, and the complex DT conditions, which can be understood as within-subject factors for the pre-intervention assessment only. In case of a violation of sphericity, Greenhouse-Geisser corrected values are reported. Univariate pairwise comparisons using Bonferroni corrected dependent *t* tests are used to uncover possible differences between the three walking conditions.

Mean differences between walking conditions are considered significant when *p* value is <0.05. The effects of the intervention on the IG were investigated by computing repeated measures MANCOVAs with the three walking conditions as within-subject factors for each of the three time points (pre-intervention, post-intervention, follow-up). The between-subjects factor was the group variable (IG or CG), with gait parameters as dependent variables. The Barthel score, maximal handgrip strength and length of stay in facility were introduced in the model as possible confounders because the effect sizes between IG and CG for all three scores were found to be substantial. Cohen’s *ds* turned around 0.70 for the Barthel score and maximal handgrip strength and around 4.6 for length of stay.

To quantify the participants’ ability to execute two tasks concurrently and to ease the interpretation of possible intervention effects, we computed DT costs for each subject according to the formula DTC = ((DT walking − normal walking/normal walking) × 100). Hence, two DTC scores were computed, (a) the simple DTC score representing DT costs between the normal walking condition (single task) and the simple DT condition, and (b) the complex DTC score representing DT costs between the normal walking condition (single task) and the complex DT condition. Given the restricted sample size of the present study, statistical power of our analyses of (co-)variances is limited. We therefore report Kazis effect sizes *δ* to quantify the amount of meaningful change in our gait parameters [21, 22]. Guidelines for interpreting an effect size are 0.2 for small, 0.5 for moderate, and 0.8 for large changes [23].

## Results

### Dual-task and gait

We find a significant main effect of the within-subjects factor walking condition on gait speed (*F*(1.3, 26.0) = 12.00, *p* = 0.001), with participants walking significantly slower in the complex DT condition (51.1 cm/s) compared to the single walking condition (59.9 cm/s). A significant effect of walking condition on the coefficient of variation of stride time (*F*(1.3, 26.8) = 6.32, *p* = 0.01) is furthermore observed, demonstrating that participants have a reduced gait stability under the complex DT condition (7.9 %) compared to the single walking condition (4.9 %). No significant differences in gait speed and stride time variabilities were observed between the single walking condition and the simple DT condition (see Table 2).

**Table 2:** Walking performances during the Three Walking Conditions with Pairwise Comparisons

		Single walking	Simple DT	Complex DT	Single walking vs. simple DT		Single walking vs. complex DT	
					Mean difference (*p* value)	95 % CI of mean difference	Mean difference (*p* value)	95 % CI of mean difference
Gait speed (cm/s)	M (SD)	59.9 (16.0)	58.8 (16.8)	51.1 (17.3)	1.1 (0.23)	−1.9; 4.1	8.9 (0.003*)	3.5; 14.2
CoV stride length (%)	M (SD)	6.9 (4.7)	6.5 (3.2)	8.6 (4.2)	0.4 (0.28)	−1.1; 1.9	−1.7 (0.10‡)	−3.7; 0.4
CoV stride time (%)	M (SD)	4.9 (3.0)	5.0 (2.4)	7.9 (4.7)	−0.2 (0.39)	−1.2; 0.9	−3.0 (0.02†)	−5.3; −0.6

*p* values based on dependent *t* test statistics, one-tailed

* Significant at the 0.01 significance level

† Significant at the 0.05 significance level

‡ Tentatively significant at the 0.10 significance level

### Cognitive training and gait

Analyses of covariance reveal no significant interaction effects on mean values between group and time of measurement on the three investigated gait parameters, gait speed, CoV stride time, and CoV stride length (ps > 0.18; see Table 3).

**Table 3:** Mean values, standard deviations, and 95 % confidence intervals of gait speed, and coefficient of variations of stride time and stride length during single walking, simple dual-task (DT) walking, and complex DT walking conditions at pre-intervention, post-intervention, and follow-up assessments for both groups (IG, CG)

Gait speed (in cm/s)		Pre-intervention		Post-intervention		Follow-up	
		Mean ± SD	95 % CI [LL; UL]	Mean ± SD	95 % CI [LL; UL]	Mean ± SD	95 % CI [LL; UL]
Single walking	IG	66.1 ± 14.6	[57.1; 75.0]	66.3 ± 16.6	[56.1; 76.5]	63.5 ± 14.8	[54.4; 72.6]
	CG	51.7 ± 15.0	[41.2; 62.3]	61.1 ± 17.0	[49.0; 73.1]	60.7 ± 15.2	[50.0; 71.5]
Simple DT walking	IG	63.0 ± 16.7	[52.7; 73.2]	62.3 ± 15.0	[53.2; 71.5]	60.5 ± 17.5	[49.8; 71.2]
	CG	53.2 ± 17.1	[41.1; 65.3]	56.5 ± 15.3	[45.7; 67.3]	56.5 ± 17.9	[43.9; 69.2]
Complex DT walking	IG	54.7 ± 19.4	[42.9; 66.6]	58.3 ± 17.0	[47.9; 68.8]	53.9 ± 17.9	[42.9; 64.8]
	CG	46.2 ± 19.8	[32.2; 60.2]	49.8 ± 17.5	[37.4; 62.1]	49.1 ± 18.3	[36.2; 62.1]

CoV stride time (in %)		Pre-intervention		Post-intervention		Follow-up	
		Mean ± SD	95 % CI [LL; UL]	Mean ± SD	95 % CI [LL; UL]	Mean ± SD	95 % CI [LL; UL]
Single walking	IG	3.8 ± 2.1	[2.5; 5.1]	3.6 ± 1.4	[2.7; 4.4]	4.4 ± 2.0	[3.2; 5.7]
	CG	6.4 ± 2.2	[4.9; 7.9]	4.1 ± 1.5	[3.1; 5.2]	5.2 ± 2.1	[3.8; 6.7]
Simple DT walking	IG	3.9 ± 2.0	[2.7; 5.1]	5.4 ± 3.1	[3.5; 7.4]	5.5 ± 3.1	[3.6; 7.4]
	CG	6.6 ± 2.0	[5.2; 8.1]	6.0 ± 3.2	[3.7; 8.2]	6.3 ± 3.2	[4.1; 8.6]
Complex DT walking	IG	8.6 ± 5.6	[5.2 12.0]	6.5 ± 3.5	[4.4; 8.6]	11.1 ± 7.0	[6.8; 15.4]
	CG	6.9 ± 5.7	[2.9; 11.0]	6.2 ± 3.5	[3.7; 8.7]	8.2 ± 7.1	[3.2; 13.3]

CoV stride length (in %)		Pre-intervention		Post-intervention		Follow-up	
		Mean ± SD	95 % CI [LL; UL]	Mean ± SD	95 % CI [LL; UL]	Mean ± SD	95 % CI [LL; UL]
Single walking	IG	4.8 ± 3.7	[2.5; 7.1]	5.5 ± 3.0	[3.6; 7.3]	5.4 ± 2.2	[4.0; 6.7]
	CG	9.8 ± 3.9	[7.1; 12.5]	5.9 ± 3.1	[3.7; 8.1]	5.4 ± 2.3	[3.8; 7.0]
Simple DT walking	IG	5.9 ± 2.3	[4.5; 7.3]	5.6 ± 1.8	[4.5; 6.7]	5.4 ± 3.7	[3.2; 7.7]
	CG	7.4 ± 2.4	[5.7; 9.0]	5.7 ± 1.8	[4.4; 7.0]	6.9 ± 3.8	[4.3; 9.6]
Complex DT walking	IG	8.2 ± 3.8	[5.9; 10.5]	6.6 ± 5.4	[3.3; 9.9]	8.3 ± 5.3	[5.1; 11.6]
	CG	9.2 ± 3.9	[6.4; 11.9]	9.3 ± 5.5	[5.4; 13.2]	10.0 ± 5.4	[6.2; 13.9]

**Fig. 2:**
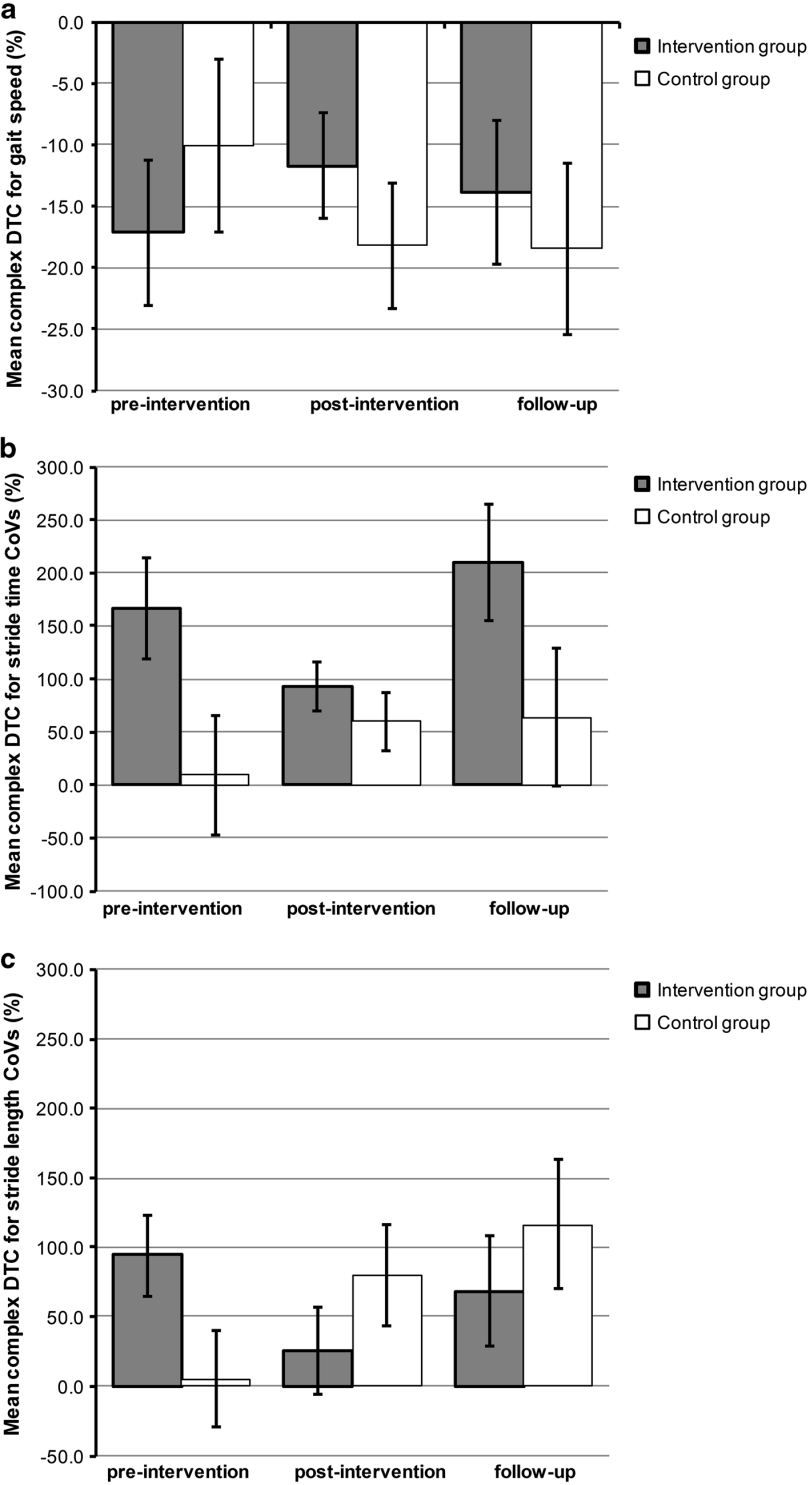
Mean dual-task costs in gait speed (**a**), Stride time variability (**b**), and Stride length variability (**c**) at pre-intervention, post-intervention, and follow-up assessments for the IG (*n* = 12) and the CG (*n* = 9). *Error bars* represent standard errors of mean

Concerning DTC in gait speed, we observe encouraging results in the expected direction with small clinically meaningful effects in the IG between pre-intervention and post-intervention assessments (*δ* = |0.26|), suggesting a less dramatic reduction of gait speed under the complex DT situation after the intervention compared to prior the intervention. This effect persists somewhat up until 3 months after the end of the intervention, with *δ* = |0.16| between pre-intervention and follow-up assessments (Fig. 2a). Computed interactions between group and time of measurement for the single walking condition compared to the simple DT condition (*F*(2,32) = 1.38, *p* = 0.27) and for the single walking condition compared to the complex DT condition (*F*(2,32) = 1.60, *p* = 0.22) were non-significant. In the CG we observe an increasing DTC during the course of the study (total study period of approximately 18 weeks).

Concerning DTC in CoV stride time, no significant interaction effects between group and time of measurement were found (ps > 0.25). Interestingly, one finding in the expected direction was observed, with our IG experiencing less complex DTC in stride time variability after the intervention (93.0 %) compared to the pre-intervention assessment (1.67 factor increase; see Fig. 2b). This change represents a small to medium-sized clinically meaningful effect (*δ* = 0.45), which is, however, unstable in time as stride time CoVs are again largely increased during the follow-up assessment (2.10 factor increase). In the CG, DTC increases from 9.3 % at the pre-intervention assessment up to 63.9 % during the follow-up assessment.

Concerning DTC in CoV stride length, no significant interaction effects between group and time of measurement were found for simple DTC (*F*(2,32) = 2.52, *p* = 0.10) and for complex DTC (*F*(2,32) = 2.34, *p* = 0.11). Although both interactions are non-significant, participants in the IG demonstrated important changes in their stride length variability at the post-intervention (16.7 and 25.6 %, respectively) assessment compared to the pre-intervention assessment (29.3 and 93.9 %, respectively), representing a small-sized clinically meaningful change for the simple DTC interaction (δ = 0.25) and a medium- to large-sized change for the complex DTC interaction (δ = 0.68; see Fig. 2c). Simple DTC is observed to be further reduced during the follow-up assessment in the IG (effect size between pre-intervention and follow-up assessments, δ = 0.54), contrary to the magnitude of the complex DTC. This suggests a limited stability over time of the observed complex DTC effect, with effect sizes between pre-intervention and follow-up assessments of δ = 0.24. Again, the CG differs from the IG at baseline with DTCs increasing dramatically over the entire study period (from 5.0 % at baseline up to a 1.17 factor increase at follow-up).

## Discussion

We found evidence for the gait-cognition association, with participants reducing their gait speed and increasing their stride time variability (reduced gait stability) significantly under the complex walking situation compared to the single walking situation. Concerning the effects of cognitive training on gait parameters, clinically meaningful improvements on the costs induced by the complex dual task were observed for gait speed and gait variabilities, with both demonstrating relative stability over a period of 3 months (although somewhat reduced).

### Dual-task and gait

The current findings confirm the existing link between cognition and gait performance (e.g., [5, 8, 9]). We found significant deteriorations of walking performance under the complex DT condition for gait speed and stride time variabilities, but not for stride length variabilities (although suggestive). The present findings corroborate previous data (e.g., [24, 25]), as gait abnormalities (and hence, fall risk) increase with the complexity of the secondary task.

### Cognitive training and gait

With the present study, we aimed at improving gait parameters experienced by old adults under complex DT situations. We obtained several results pointing in the expected direction. We observed small to large clinically meaningful changes in the IG after the training with improved gait parameters under complex DT situations. This suggests that participants in the IG experience a weaker negative influence of the complex cognitive task on their gait performance after the intervention. The present data corroborates previous findings [11] on gait speed under normal and walking-while-talking situations. The IG of Verghese and colleagues (composed of community-dwelling older adults) improved their gait speed under the walking-while-talking situation by 4 cm/s, representing a small but clinically meaningful change [22]. Similar changes were observed in our IG with a mean gait speed improvement of 3.6 cm/s (see Table 3) and thus extending Verghese’s findings to a group of old and frail nursing home residents. 3 months after the intervention, two meaningful effects remained, (a) improvements in gait speed and (b) increase in stability of stride length under the simple DT. It is unclear if the instability of the improvements in stride time and stride length (under the complex DT situation) are due to specific characteristics of the present sample or the cognitive training program. Research focusing on long-term training effects will need to disentangle these factors. A recent study which implemented a 12-month cognitive training program observed relatively large training gains, but they appeared only after 9 months of the program and remained constant for the following 6 months [26]. Hence, this may indicate that increasing the duration of cognitive training programs may lead not only to larger but also to more stable effects in time.

Given the pilot nature of the present study, our investigation demonstrates a number of limitations. First, the restricted sample size limits us from producing clear-cut conclusions and prohibits us from generalizing the present findings to a larger population. An anonymous reviewer suggested that we increase our sample size; while we completely agree that large samples sizes from clinical populations are highly desirable, we contend that small sample studies (as this one) in an emerging field of research are crucial for spurring future research and clinical studies. Second, some baseline gait parameters in the IG differed from parameters in the CG (CoVs). It remains unclear to what factors these baseline differences in gait parameters are attributable, especially as investigations of gait were performed for both groups according to the exact same protocol with the same investigator and gait data were double-checked for errors. Note that both groups did not significantly differ in terms of their overall characteristics (see Table 1). Although controlling a posteriori for several possible influences by means of covariates, the remaining baseline differences are likely to be attributable to our not completely randomized samples. More precisely, it can be said that the (quasi-) randomization of our samples was biased by patients who were not willing to participate in the IG, but agreed to participate as a control in the study. This shortcoming may have influenced the present data in an unforeseen and non-random manner. Hence and because of this bias, we are unable to account for all possible a priori differences between groups, i.e., unobserved heterogeneity that was not taken into account in the present study. Taken together, these limitations prohibit us from concluding definitively on the positive influence of the cognitive training program on gait, but our results are suggestive of such a claim. Thus, following the present pilot study, we have created a larger interdisciplinary research group that aims at studying more precisely the effects of structured cognitive training programs on different levels (e.g., cognitive and functional level) in older adults, using a number of different research methods.

## Conclusion

The present pilot study’s approach of improving gait under challenging walking situations by interventions designed to improve cognition adds encouraging results to this promising field of research. Importantly, findings of the present study go in the expected direction suggesting that cognitive intervention effects may transfer to an untrained gait domain. Our data corroborates previous findings (see gait-cognition interaction, DT complexity) and extends them to the group of frail old nursing home residents. In conclusion, the data, although preliminary, is encouraging and thus warrants further investigations.
